# Betalain exerts a protective effect against glaucoma is majorly through the association of inflammatory cytokines

**DOI:** 10.1186/s13568-020-01062-y

**Published:** 2020-07-14

**Authors:** Jiadi Wang, Dandan Zhang, Conghong Cao, Jing Yao

**Affiliations:** Department of Ophthalmology, Heilongjiang University of Traditional Chinese Medicine, No 26 Heping Road, Xiangfang District, Harbin, 150040 Heilongjiang China

**Keywords:** Betalain, Inflammatory, Glaucoma, Cytokines

## Abstract

The present research aimed at evaluating the protective role of betalain on the in vitro glaucoma model using PC12 neuronal cells. The cultured neuronal cells in a customized pressurized chamber were analyzed for the onset of glutathione, myeloperoxidase (MPO), cathepsin, expression of inflammatory enzymes such as cyclooxygenase (COX-1), lipoxygenase (5- LOX), sPLA2 caveolin-1, glaucoma markers and other inflammatory cytokines in the presence and absence of betalain. The results have shown that a significant increase in the expression of oxidative stress with increased activity of cathepsin B and D. On the other hand, the activity of inflammatory enzymes such as COX-1, 5- LOX, sPLA2 were significantly increased in pressure exposed cells. In addition, glaucoma simulated cells demonstrated a significant increase in the VEGF, TGF-β, BDGF, and neuroserpin compared to control. Moreover, cells predisposed to hydrostatic pressure demonstrated an increase in (p < 0.01) inflammatory cytokines such as IL-6, CXCR4, IL-17, IL-1β, and TNF-α levels. However, cells pre-treated with betalain improved the glutathione levels with attenuated MPO activity. Simultaneously, the levels of inflammatory cytokines and other glaucoma marker genes found restored in drug pre-treated cells. Thus, the results of the present study demonstrate that the use of betalain on ocular cells can prevent the progression of the disease that can be a suggestive therapeutic for controlling glaucoma like conditions.

## Keypoints

- The protective role of betalain on the in vitro glaucoma model using PC12 neuronal cells. They were analyzed for the onset of glutathione, myeloperoxidase, cathepsin, expression of inflammatory enzymes.
- Glaucoma simulated cells demonstrated a significant increase in the VEGF, TGF-β, BDGF and neuroserpin compared to control.
- Cells pre-treated with betalain improved the glutathione levels with attenuated MPO activity. The levels of inflammatory cytokines and other glaucoma marker genes were restored in drug pre-treated cells.

## Introduction

The optic neuropathy disease, glaucoma is defined by the loss of retinal ganglion cells and their axons that are complicated by the optic nerve head, and retinal tissue remodeling (Smith et al. 2017) is progressive and multifactorial (Shahsuvaryan 2013) and is debilitating in the old age due to the blindness of the retina. Glaucomatous optic neuropathy takes time to develop, and hence the disease is known to be progressive. Patients suffering from this disease would be having difficulty in vision with a great decrease in the contrast and color sensitivity (Bambo et al. 2016). The worldwide cases of glaucoma are on the increase due to poor practices in handling electronics gadgets, changed life style, causing tremendous pressure on the healthcare system of the world due to the health expenses and loss of revenue for the patients.

Glaucoma is known to be associated with an increase in the intraocular pressure (IOP) and is considered to be one of the pathological features of glaucoma although various cases have debunked the correlation between IOP increase and development of glaucoma (Mozaffarieh and Flammer 2007). Elevation of IOP compresses the retinal ganglion axons and affects the axonal transport for the delivery of nutrition to the survival of ganglions (Ster et al. 2014). This may onset a mild reperfusion injury that would ensure inflammation and accelerate the production of free radicals and results in oxidative damage (Renner et al. 2017). Animal models have shown that such reperfusion injury has elevated the intraocular pressure transiently, and that would induce necrosis or apoptosis in the ganglions (Renner et al. 2017). Ganglions may undergo apoptosis and results in the death of these cells and causes glaucomatous optic neuropathy in which oxidative stress has been implicated (Oharazawa et al. 2010). Oxidative stress results in the imbalance in the antioxidant defenses to the levels of reactive oxygen species generated and has been suggested to cause retinal ischemic injury and in glaucoma pathogenesis (Kortuem et al. 2000; Kuriyama et al. 2001).

Betalains are water-soluble nitrogenous pigments that are naturally occurring compounds that give colours to plants and have strong antioxidant activity (Miguel 2018). The health-promoting activities of betalains have been extensively studied in the context of their anticancer, anti-inflammatory, hepatoprotective and have been commercialized as dietary supplements and used in food colorants in the food industry (Kaur et al. 2018). The antioxidative capacity of betalain has been studied extensively in the context of scavenging the free-radicals, and the blood pressure-lowering capacity of betalain on the nervous system (Rahimi et al. 2019) has tempted us to look into these characteristics in reducing the complications of glaucoma that are induced due to increased pressure on the retinal nerves in causing apoptosis of the ganglion cells.

In the course of study, we have used PC12 cells and culturing them in a customized pressurized chamber to mimic the conditions of ocular pressure as in glaucoma and analyzed for the expression of inflammatory markers, pro-oxidation markers in the extent of oxidative damage and treating the cells with betalain to observe the recovery of PC12 cells from the mechanized pressure which would help in extending the results to in vivo model of glaucoma for the use of this as a novel candidate in treatment.

## Materials and methods

### Cell culture and induction of glaucoma

PC12 cell line, the neuronal cells for the experimental model for ocular diseases were obtained from the American Type Culture Collection (ATCC, Virginia, USA). The cells were grown in Dulbecco’s modified Eagle’s Medium (DMEM) supplemented with 10% fetal bovine serum, 100 units/ml of penicillin, and 75 μg/ml of streptomycin. The cells were maintained at 37 °C in a humidified incubator containing 95% air and 5% CO_2_ for maintenance. For the induction of glaucoma elevations of pressure represent the gold standard model of ocular hypertension. A customized pressurized chamber was employed in the study which injects a mixture of humidified gas (95% air/5% CO_2_) through a pressure regulator which maintains the stable pressure within ± 1 mm Hg from 0 to 200 mm Hg, and it is continuously regulated using a pressure gauge (Ju et al. 2007; Tok et al. 2014). For the experimentation, 50% confluent cells in culture plates and flasks were grown with or without pressure and betalain drug (75 µM) for 24 h. At the end of the experimental period, the cells were scraped off and used for various biochemical analyses and gene expression studies.

### Enzyme and inflammatory enzymes

For the assessment of the cellular status of enzymes such as glutathione (ab65322) cathepsin B (ab65300), cathepsin D (ab65302), myeloperoxidase (MPO) (ab105136), commercial enzyme assay kits were used and the assays were performed as per the manufacturer’s protocol (Abcam Inc, Massachusetts, USA). To elucidate the inflammatory responses, the marker enzymes such as cyclooxygenase (COX-2, (ab204699), lipooxygenase (5- LOX, ab241038), sPLA2 and leukotriene B4 (Cayman Chemicals, USA) were estimated in the cells using commercial assay kits (Wuhan Fine Biotech Co., Ltd., Hubei, China).

### Cytokine and ocular protein level estimation

To elucidate the role of cytokines in glaucoma, cells exposed to pressure and drugs along with control were assayed for the pro-inflammatory markers such as IL-17, CXCR4, IL-6, IL-1β, and TNF-α using commercial kits as per the manufacturer’s instruction (Fine Biotech Co., Ltd, Hubei, China). Moreover, the levels of optineurin (OPTN), beta-Arrestin-1, nestin, connexin 43, VCAM-1, ICAM-1 were also analyzed in the control and experimental cells using the commercial assay kits (Abcam Inc, Massachusetts, USA).

### Reverse transcription-PCR

The marker genes of glaucoma onset and related signaling genes were analyzed using qRT-PCR. The total RNA was extracted from the cells using TRIzol reagent. The cell suspension along with TRIzol was incubated at room temperature for 5 min, and an equal volume of chloroform was added, mixed vigorously and centrifuged at 15,000*g* for 10 min at 4 °C. The upper aqueous phase was collected, and an equal volume of isopropanol was added and incubated for 20 min at 4 °C and centrifuged at 12,000*g* for 20 min at 4 °C. The obtained RNA pellet obtained was washed with 70% ethanol and quantified using Spectrophotometer. For the cDNA synthesis, an equal amount of RNA from all groups was transcribed to cDNA (Qiagen China Shanghai Co Ltd, Shanghai, China), and the real-time RT-PCR was done for specific genes using SYBY green/ROX master mix and amplified using the Roche real-time PCR system. The gene expression was calculated from the CT values, and the fold increase was determined by the comparative Ct method (ΔΔCT) with expression values of GAPDH as the endogenous control. The forward (F) and reverse (R) primers used for the specific genes were given in Table 1.

**Table 1:** Primer used in our study

Gene	Primer	Sequence (5′–3′)	Annealing
VEGF	F	CAAACCTCACCAAAGCCAGC	59
	R	GCGCTTTCGTTTTTGACCCT	
BDNF	F	GTGGTTACCTGACTGGGCTC	58
	R	TCCCTGAGTCACAGTGGACA	
Caveolin-1	F	GCGCCTTTCCCCCTCTATAC	57
	R	GGGCTTGTAGATGTTGCCCT	
TGF-β	F	CCACGTGGAAATCAATGGGA	59
	R	TGCCGTACACAGCAGTTCTT	
Myocilin	F	TACCTCGGGGCTTGTACAGT	58
	R	CCATACAGCTTGGCAAAAAGCA	
Neuroserpin	F	GCTGTGGCCTCAGGAATGAT	58
	R	GTGCATGACTCGTCCCATGA	
GAPDH	F	TCTCTGCTCCTCCCTGTTCT	57
	R	TACGGCCAAATCCGTTCACA	

### Statistical analysis

GraphPad Prism Software (GraphPad Software, San Diego, CA) was used for the analysis of the study results. The data are expressed as mean ± standard error (SE). The *p* value of < 0.05 was considered significant.

## Results

**Fig. 1:**
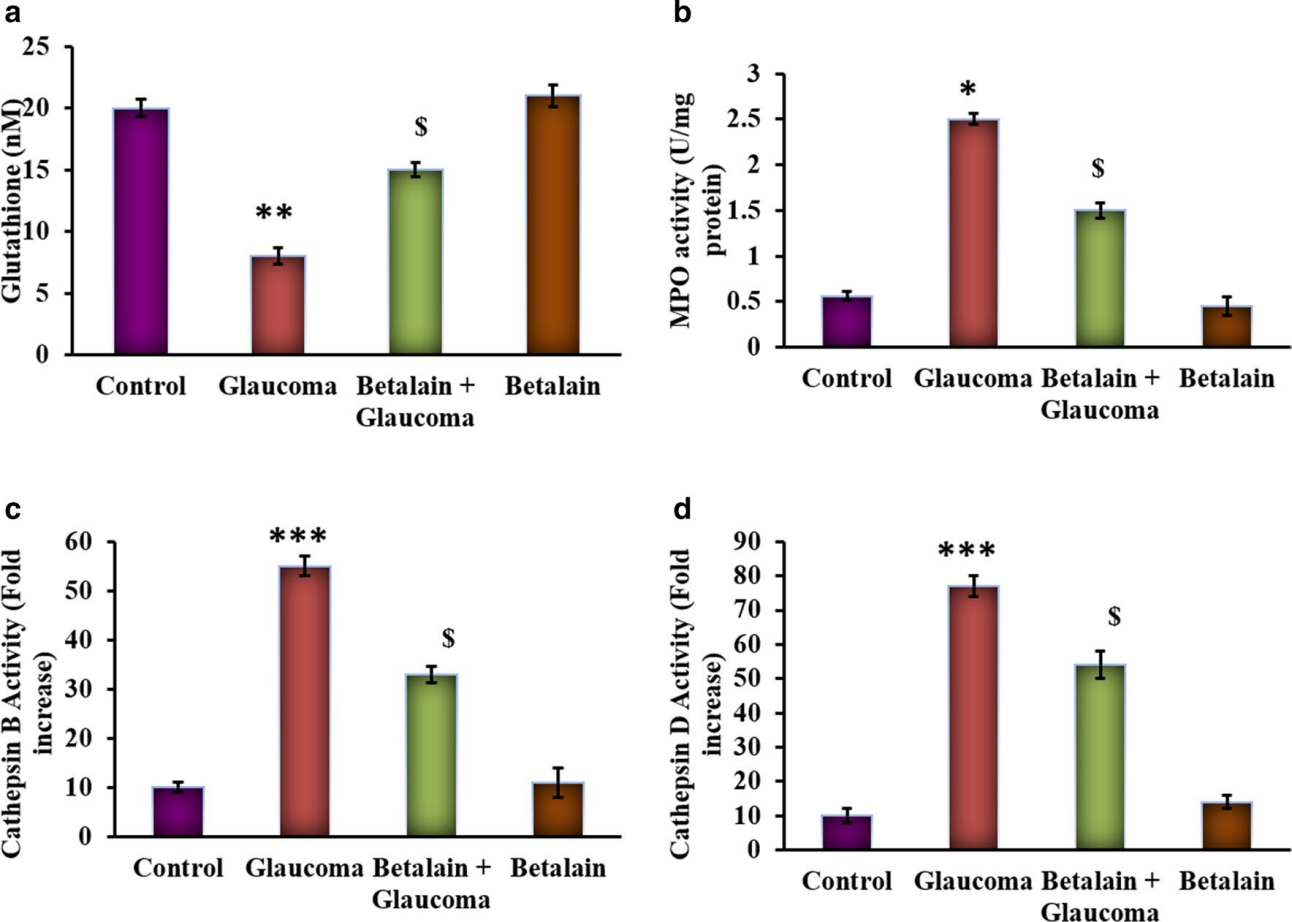
**a**–**d** represents the levels of glutathione, myeloperoxidase (MPO), cathepsin B, cathepsin D in the control and experimental cells. Values are expressed as mean ± SE (n = 12). Statistical significance expressed as *p < 0.05, **p < 0.01, ***p < 0.001 compared to untreated controls, ^$^p < 0.05 betalain + Glaucoma exposed cells compared with glaucoma simulated cells

The current investigation was performed to evaluate the protective effect of betalain against the onset of glaucoma. PC12 neuronal cells were cultured and maintained in hydrostatic pressure conditions and tested for the cellular inflammatory responses. Initially, the PC12 cells grown under hydrostatic pressures demonstrated the increased (p < 0.01) MPO with reduced levels of glutathione compared to control cells grown under no pressure. Besides, the levels of cathepsin B and D were significantly raised in cells displayed glaucoma like symptoms. However, cells that had drug treatment earlier displayed a significant reduction in its level compared to cells had pressure exposure (Fig. 1).

**Fig. 2:**
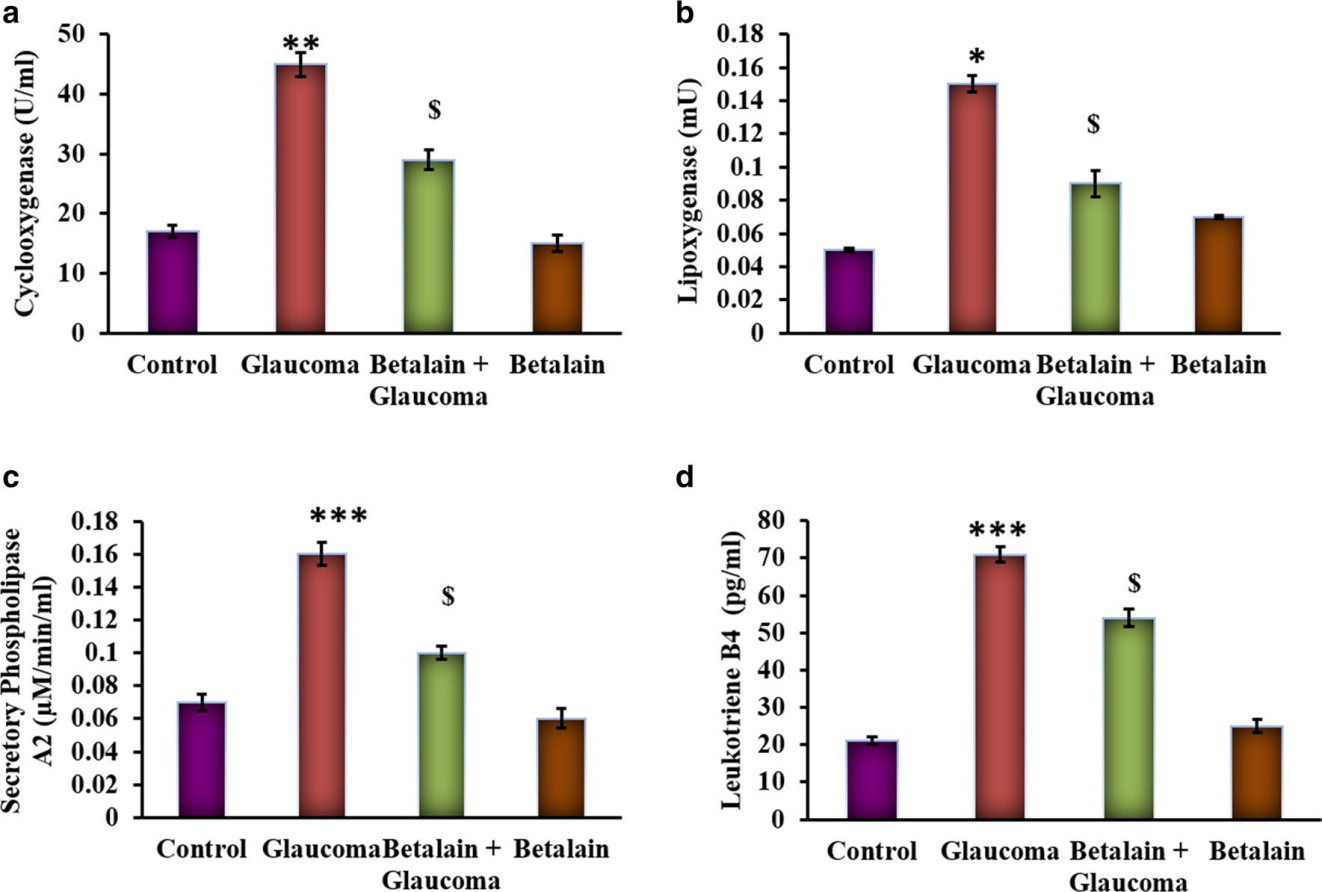
**a**–**d** represents the activity of inflammatory enzymes in the control and experimental cells. The details of the methodology were given in the experimental section. Values are expressed as mean ± SE (n = 12). Statistical significance expressed as *p < 0.05, **p < 0.01, ***p < 0.001 compared to untreated controls, ^$^p < 0.05 betalain + Glaucoma exposed cells compared with glaucoma simulated cells

It is well demonstrated that the inflammatory mediators play a major role in progressing the cells towards damage. In the present study, glaucoma simulated cells evinced (p < 0.01) an increase (p < 0.05) in the activities of cyclooxygenase (COX-2), lipoxygenase (5- LOX), sPLA2, leukotriene B4 compared to control cells. However, cells exposed to betalain reduced the levels of these enzymes to near normalcy (Fig. 2) shows the protective effect of betalain.

**Fig. 3:**
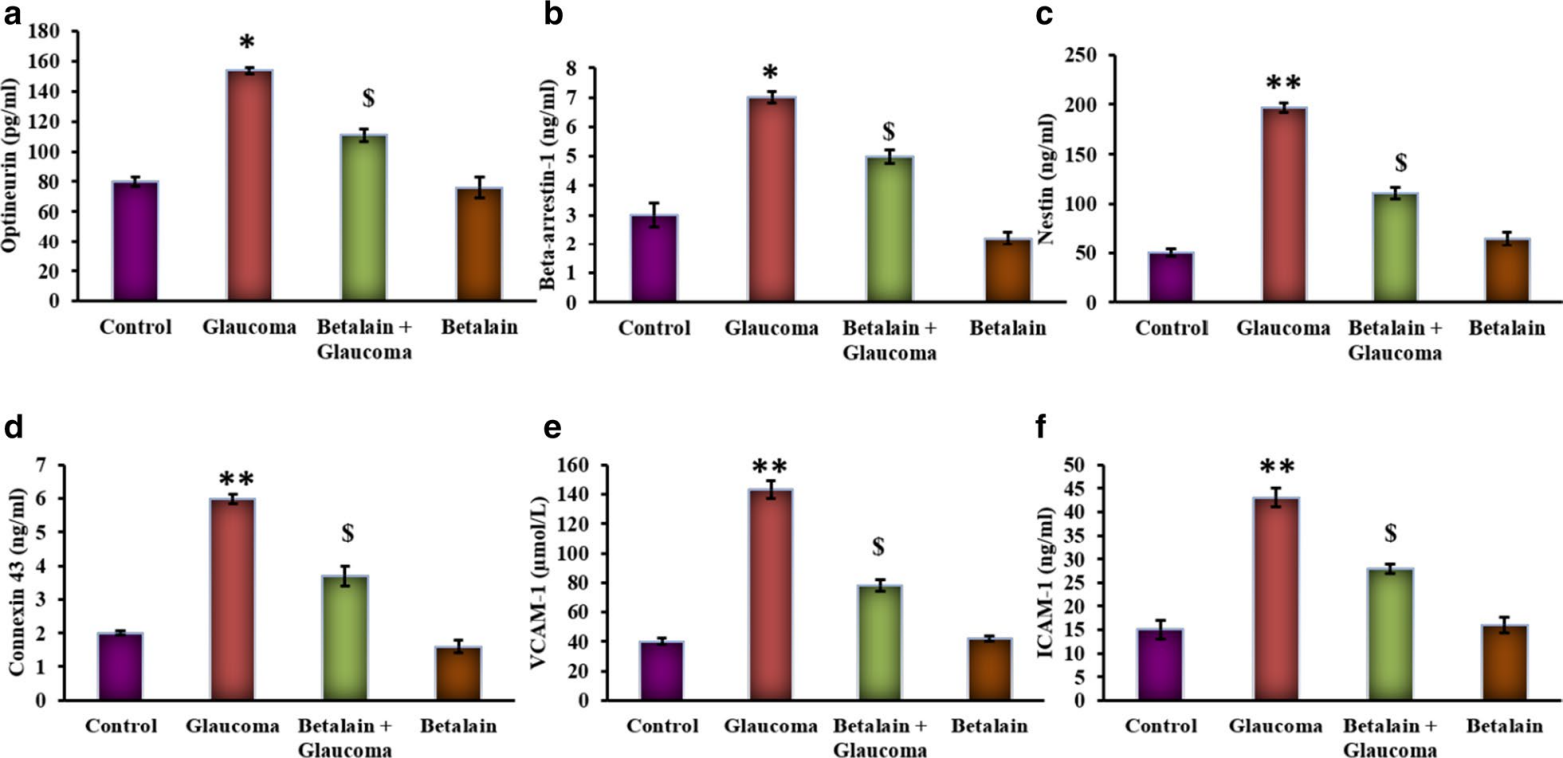
**a**–**f** represents the OPTN, β-Arrestin-1, nestin, connexin 43, VCAM-1, and ICAM-1 levels in the control and experimental group of cells. Values are expressed as mean ± SE (n = 12). Statistical significance expressed as *p < 0.05, **p < 0.01 compared to untreated controls, ^$^p < 0.05 betalain + Glaucoma exposed cells compared with glaucoma simulated cells

Figure 3 shows the levels of ocular derangement proteins in the control and experimental group of cells. Cells underwent pressure treatment mimicking the glaucoma condition displayed a significant (p < 0.05) increase in optineurin (OPTN), beta-Arrestin-1, nestin, connexin 43, VCAM-1, and ICAM-1 proteins compared to control, while the cells had a pre-exposure to betalain exhibited the reduced levels of these deranged proteins suggest that the drug acts on the signaling and curtail them which are appealing towards malfunctioning.

**Fig. 4:**
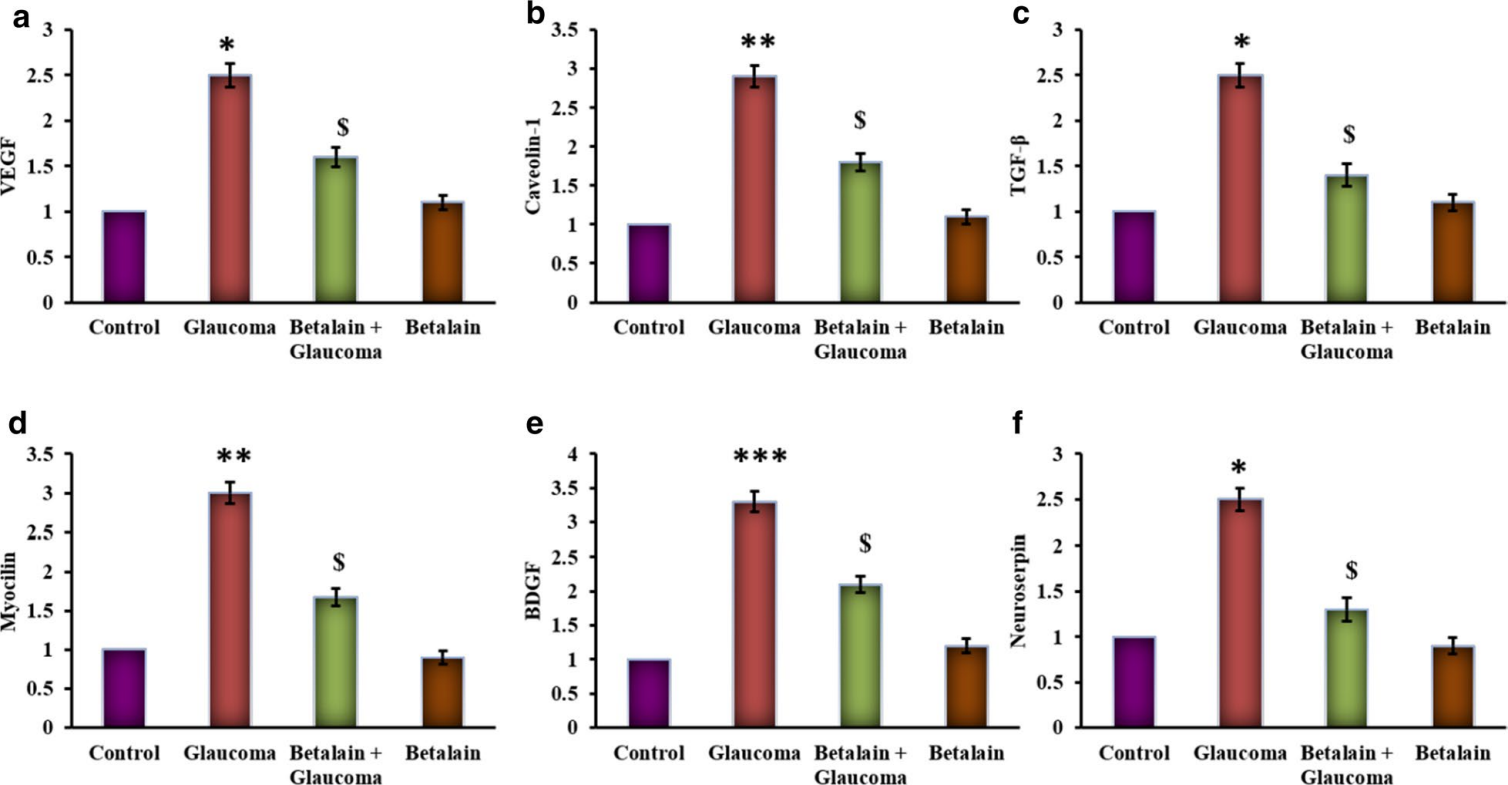
**a**–**f** represents the qRT-PCR mRNA expression analysis of ocular marker genes of control and experimental group of cells. Values are expressed as mean ± SE (n = 12). Statistical significance expressed as *p < 0.05, **p < 0.01, ***p < 0.001 compared to untreated controls, ^$^p < 0.05 betalain + Glaucoma exposed cells compared with glaucoma simulated cells

Besides, marker genes of glaucoma and the related growth responsive genes were elucidated using RT-PCR, and the results are presented in Fig. 4. The results substantiated that the expression of VEGF, BDNF, Caveolin-1, TGF-β, Myocilin, and neuroserpin genes were increased (p < 0.05) compared to control. While the betalain-exposed cells displayed an admissive decrease in the marker genes compared to glaucoma simulated group delineate the protective mechanism activated by the drug against glaucoma (Fig. 4).

**Fig. 5:**
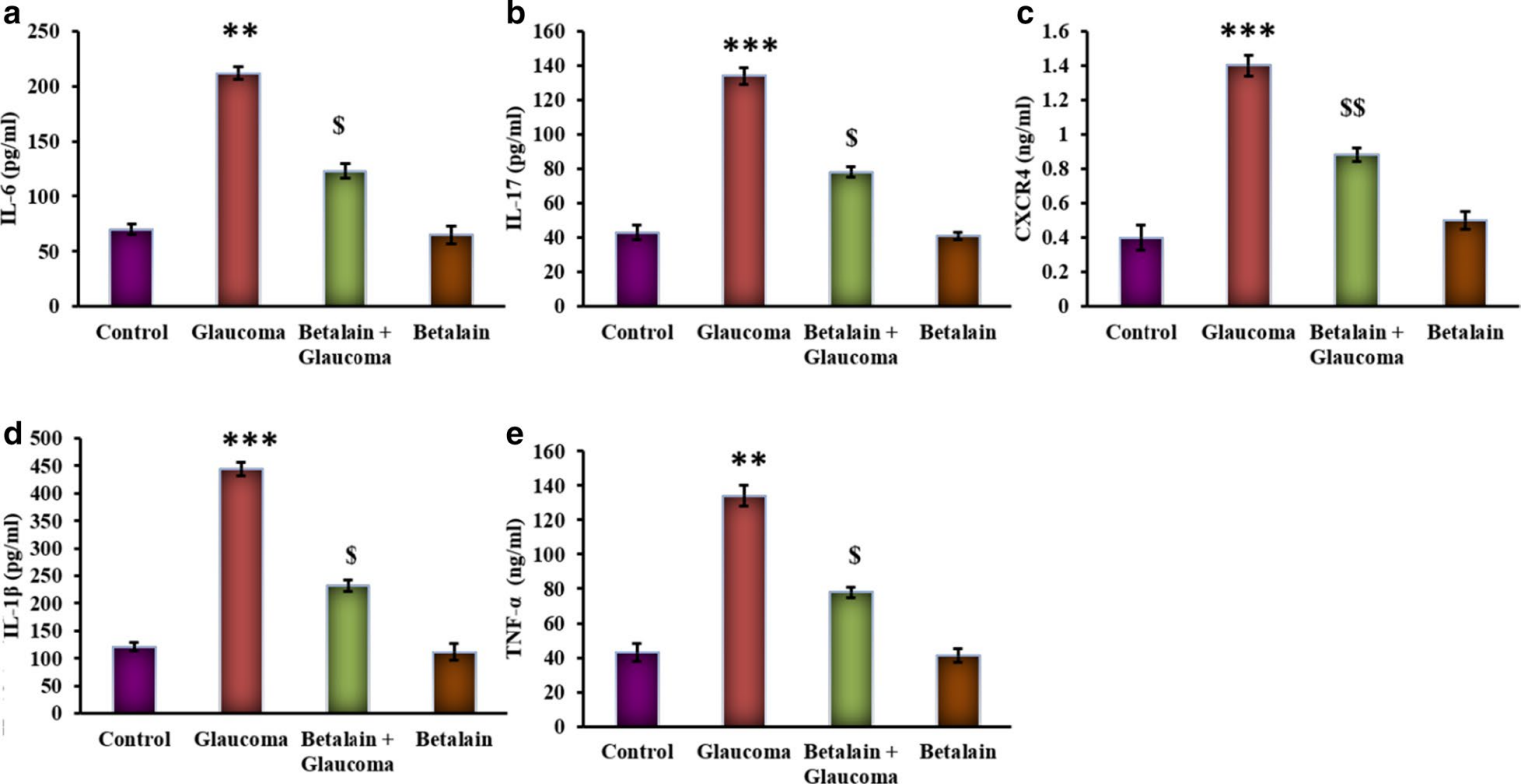
**a**–**e** represents cytokine expression analysis of IL-6, CXCR4, IL-17, IL-1β, and TNF-α in the control and experimental group of cells. Values are expressed as mean ± SE (n = 12). Statistical significance expressed as **p < 0.01, ***p < 0.001 compared to untreated controls, ^$^p < 0.05 betalain + Glaucoma exposed cells compared with glaucoma simulated cells

Figure 5 represents the levels of cytokines in cells with or without drug and pressure exposure. Cells grown under hydrostatic pressure displayed a prominent (p < 0.05) increase in the levels of cytokines such as IL-6, CXCR4, IL-17, IL-1β, and TNF-α compared to control. While these inflammatory cytokines were reduced in betalain treatment point out that the glaucoma progression is attenuated by betalain via suppressing cytokines (Fig. 5).

## Discussion

Elevated intraocular pressure is considered to be an important factor in the onset of glaucoma and it causes compression of the retinal ganglion cells that obstruct the flow of blood and nutrition to the cells and that result in temporary retinal reperfusion injury (Kwon et al. 2009). This injury would cause inflammation in the retinal cells and is measured by the increase in the inflammatory markers. In our PC12 glaucoma model, the increase in oxidative stress may increase nitric oxide, which is an important mediator of the blood flow to the retina and helps ganglion cells to get nourishment. They are observed to be increased in glaucoma and diabetic patients (Hangai et al. 1999; Nathanson and McKee 1995). Associated with that, the levels of MPO, which is an inflammatory enzyme and known to be expressed during ischemia (Loria et al. 2008), have increased (Lin et al. 2005) for the generation of free radicals to generate the reactive nitrogen species (Eiserich et al. 1996, 1998) to overwhelm the oxidative damage on the retinal cells.

Protection to the eye in glaucoma patients has been conferred by antioxidant enzyme glutathione (Gherghel et al. 2005) which prevents the effects of ROS by maintaining the cellular antioxidants higher (Bendich 1990; Pompella and Corti 2015). Such levels have decreased in the glaucoma patients as in our PC12 cells in hydrostatic pressure conditions. Recovery of these cells have happened with the treatment of the cells with betalain which has the antioxidant capacity to target the oxidative stress (Masuda et al. 2017) increase the levels of glutathione to normal levels and decreased the MPO enzymes. The accumulated ROS would induce the lysosomal membrane to release the Cathepsin B and D to degrade the TM cells and cause cell death to increase the IOP (Zhang et al. 2017) which in otherwise have maintained in the anterior ocular region (Lin et al. 2010). Cathepsins B and D that follow the lysosomal pathway for the digestion of the trabecular meshwork cells (Porter et al. 2013) that helps maintain the IOP have increased in the PC12 cells.

In the maintenance of IOP, the COX-2 enzyme would increase its activity to increase the prostaglandins that are the prime mediators of IOP regulation. Hence its activity was increased in the PC-12 cells under hydrostatic pressure (Marshall et al. 2004) with increased activity of COX-2, an indicator of the inflammation in the retina (Iwabe et al. 2010). However, the levels were decreased in similar conditions of glaucoma in human cases (Maihofner et al. 2001). Similarly, the other mediators of inflammation such as lipooxygenase, sPLA2 (Ronkko et al. 2007) and leukotriene B4 (Sasaki et al. 2018) have seen their activity and levels decreased after treatment of the cells with betalain due to the anti-inflammatory effect of this molecule in addition to the reduction of IOP (Clifford et al. 2015).

The IOP has been found associated with upregulated expression of nestin that is an indicator of the retina injury in experimentally induced glaucoma in the glial cells (Xue et al. 2006). Further, the damage due to oxidative stress will be primarily seen in the mitochondria of the retinal cells, and hence their clearance is observed in the retina with the damaged ones by autophagy mechanism by optineurin (Swarup and Sayyad 2018) and beta-arrestin (Nicolas-Leveque et al. 1999). Alteration in the expression of the gap junction protein in the astrocytes has been the characteristic feature of acute injury in the ischemic injury, injury in the astrocytes and retina in glaucoma. It suggests a pathogenic role of connexin in glaucoma (Kerr et al. 2011) and hence targeting them would be a good target for glaucoma treatment (Sena and Lindsley 2017). The injuries in the retina and the damage to the trabeculae cells have thus seen by a greater turnover of VCAM-1 and ICAM-1 proteins and myocilin mRNA (Resch and Fautsch 2009) indicating that they are let off from the meshwork (Dong et al. 2011). Betalain treatment has ameliorated the retinal injury and decreased the expression of these proteins that are highly expressed during the complication arising out of it in case of glaucoma.

The rise in VEGF mRNA has been observed in patients who have recent retinal ischemia which would spread to the anterior of the retina and cause neo-vascularization to increase the IOP (Sun et al. 2016). The seriousness of the retinal injury can be assessed by the increase in BDNF that does not express in the early stages of glaucoma (Shpak et al. 2018). Other mRNAs that are expressed to improve the conditions of retinal injury, caveolin-1, and neuroserpin genes were found increased that are indicators of the mechanoprotection of the system (Elliott et al. 2016). Betalain offers anti-apoptotic protection and rescues the cells by reversing to the mitochondrial respiration suggest the protective effect against glaucoma.

In conclusion, reduction in IL-6 would help in reducing the effects of glaucoma as with the other inflammatory cytokines of CXCR4, IL-17, IL-1β, and TNF-α which are known to be increased in PC-12-glaucoma model cells without betalain treatment. While the trend is reversed with the cells aiming for recovery from the hydrostatic pressure effect of glaucoma with betalain. Hence the present study suggests that the drug candidate betalain is a promising candidate to be taken to the next step of research in the animal models of glaucoma to be used. This could throw light on the probable molecular pathway taken up by the physiological system in averting the effects of glaucoma.

## Data Availability

All data are fully available.
